# Supplementary material for: Gut microbiota and fecal short chain fatty acids differ with adiposity and country of origin: the METS-microbiome study
Source: Nat Commun. 2023 Aug 24;14:5160. doi: 10.1038/s41467-023-40874-x (PMC10449869; doi:10.1038/s41467-023-40874-x)
Supplement: Supplementary file 1 — Supplementary Information [file 41467_2023_40874_MOESM1_ESM.pdf]

Supplementary Information

Gut microbiota and fecal short chain fatty acids differ with adiposity and country of origin: The METS-Microbiome Study

Gertrude Ecklu-Mensah[1*], Candice Choo-Kang[2*], Maria Gjerstad Maseng[3,4,5], Sonya Donato[1], Pascal Bovet[6, 7], Bharathi Viswanathan[7], Kweku Bedu-Addo[8], Jacob Plange-Rhule[8], Prince Oti Boateng[8], Terrence E. Forrester[9], Marie Williams[9], Estelle V. Lambert[10], Dale Rae[10], Nandipha Sinyanya[10], Amy Luke[2], Brian T. Layden[11, 12], Stephen O'Keefe[13], Jack A. Gilbert[1**], Lara R. Dugas [2, 14**]

[1]Department of Pediatrics, Center for Microbiome Innovation, University of California San Diego, La Jolla, CA, USA
[2] Public Health Sciences, Parkinson School of Health Sciences and Public Health, Loyola University Chicago, Maywood, IL, USA
[3] Institute of Clinical Medicine, Faculty of Medicine, University of Oslo, Oslo, Norway
[4] Dep. of Gastroenterology, Oslo University Hospital, Oslo, Norway
[5] Bio-Me, Oslo, Norway
[6] University Center for Primary Care and Public Health (Unisanté), Lausanne University Hospital, Lausanne, Switzerland
[7] Ministry of Health, Victoria, Republic of Seychelles
[8]Department of Physiology, SMS, Kwame Nkrumah University of Science and Technology, Kumasi, Ghana
[9]Solutions for Developing Countries, University of the West Indies, Mona, Kingston, Jamaica
[10]Research Unit for Exercise Science and Sports Medicine, University of Cape Town, Cape Town, South Africa
[11]Department of Medicine, University of Illinois at Chicago, Chicago, Illinois, USA
[12]Jesse Brown Veterans Affairs Medical Center, Chicago, Illinois, USA.
[13]Department of Medicine, University of Pittsburgh, PA, USA
[14]Division of Epidemiology and Biostatistics, School of Public Health, Faculty of Health Sciences, University of Cape Town, Cape Town, South Africa.

Supplementary Table 1. Alpha diversity between obese and non-obese groups

Country	non-Obese	Obese	Observed ASVs	Shannon	Faith's PD
			P	P	P
Ghana	243	89	0.0439	0.268	0.0393
South Africa	233	141	0.0488	0.0686	0.12
Jamaica	208	179	0.338	0.494	0.239
Seychelles	217	147	0.206	0.752	0.186
USA	112	195	0.759	0.246	0.901

Alpha diversity estimated by Shannon, Observed ASVs and Faith's PD (Phylogenetic Diversity) between obese and non-obese in each country (Wilcoxon rank sum test; two-sided). Statistical significance adjusted for multiple comparisons using false discovery rate (FDR) correction are presented. Statistical significance, $p < 0.05$. n= 1764 samples. Source data are provided as a Source Data file. Exact p-values are provided.

Supplementary Table 2. Gut microbiota Beta Diversity

	Weighted UniFrac Distance											
	Overall		Ghana		South Africa		Jamaica		Seychelles		USA	
	R^2	P	R^2	P	R^2	P	R^2	P	R^2	P	R^2	P
Obese	0.001	0.031	0.004	0.208	0.003	0.415	0.002	0.732	0.001	0.903	0.007	0.043
Sex	0.003	0.001	0.002	0.549	0.004	0.19	0.009	0.009	0.023	0.001	0.011	0.004
Age	0.002	0.001	0.101	0.815	0.08	0.737	0.102	0.062	0.053	0.894	0.098	0.283
Country	0.118	0.001										
	Unweighted UniFrac Distance											
	overall		Ghana		South Africa		Jamaica		Seychelles		USA	
	R^2	P	R^2	P	R^2	P	R^2	P	R^2	P	R^2	P
Obese	0.003	0.001	0.004	0.032	0.007	0.002	0.002	0.732	0.003	0.279	0.004	0.154
Sex	0.003	0.001	0.005	0.018	0.007	0.002	0.009	0.009	0.01	0.001	0.01	0.001
Age	0.001	0.135	0.113	0.471	0.083	0.708	0.102	0.062	0.063	0.576	0.094	0.252
Country	0.083	0.001										

Adjusted multivariable analysis in the entire cohort (overall) and by each country. Statistical significance from permutational multivariate analysis of variance (PERMANOVA, two-tailed test, $p < 0.05$). n= 1764 samples. All p-values are generated based on 999 permutations and exact p-values are provided.

Supplementary Table 3 – Description of study participants by microbial enterotypes

	Bacteroides type	*Prevotella* type	p-value	q-value
N	820	866		
BMI	29(25,35)	28(23,34)	0.003	0.004
Country			<0.001	<0.001
Ghana	61 (7.4%)	265(31%)		
Jamaica	243(30%)	117(14%)		
Seychelles	149(18%)	176(20%)		
South Africa	142(17%)	232(27%)		
USA	225(27%)	76(8.8%)		
Sex	564(69%)	503(58%)	<0.001	<0.001
Age	44(38,50)	42(36,48)	0.032	0.032
Obese	370(45%)	346(40%)	<0.001	<0.001
Total SCFA	3547(2325,4891)	5300(3591,7672)	<0.001	<0.001
Bacteroides	1299(315,7521)	92(18,526)	<0.001	<0.001
Prevotella	98(25,319)	1246(198,10382)	<0.001	<0.001

Data are presented as median (interquartile range) for continuous variables and percentages (%) for categorical variables. n= 1686 samples. p values are based on Wilcoxon rank-sum test or Pearson's Chi-squared test (two-tailed). Exact q values are shown. Source data are provided as a Source Data file. Statistical significance, q < 0.05. q = False Discovery Rate correction, BMI = Body Mass Index, SCFA = short chain fatty acids

Supplementary Table 4. Confusion matrix table showing the average prediction accuracy of gut microbiota in estimating the different countries using a Random Forest model

	Prediction label					
	Ghana	South Africa	Jamaica	Seychelles	USA	Overall accuracy
Ghana	0.88	0.03	0.02	0.05	0.03	
South Africa	0.03	0.77	0.04	0.13	0.03	
Jamaica	0	0.1	0.88	0	0.03	
Seychelles	0.03	0.03	0.06	0.82	0.06	
USA	0	0.03	0.07	0.05	0.85	
Overall accuracy						0.84
Baseline accuracy						0.22
Accuracy ratio						3.79

n=1694

Supplementary Table 5. Confusion matrix table showing the average prediction accuracy of gut microbiota in a Random Forest model

	Predicted label		
	Diabetic	Non-Diabetic	Overall accuracy
Diabetic	0	1	
Non-Diabetic	0	1	
Overall accuracy			0.89
Baseline accuracy			0.89
Accuracy ratio			1
	Elevated glucose	Normal glucose	Overall accuracy
Elevated glucose	0	1	
Normal glucose	0.01	0.99	
Overall accuracy			0.75
Baseline accuracy			0.76
Accuracy ratio			0.99
	Hypertensive	Non-Hypertensive	Overall accuracy
Hypertensive	0.67	0.33	
Non-Hypertensive	0.47	0.53	
Overall accuracy			0.6
Baseline accuracy			0.5
Accuracy ratio			1.2
	Obese	Non-Obese	Overall accuracy
Obese	0.38	0.62	
Non-Obese	0.19	0.81	
Overall accuracy			0.63
Baseline accuracy			0.58
Accuracy ratio			1.09
	Female	Male	Overall accuracy
Female	0.94	0.06	
Male	0.7	0.3	
Overall accuracy			0.7
Baseline accuracy			0.63
Accuracy ratio			1.11

Prediction accuracy for estimating diabetes status (n=1657); glucose status (n=1657); hypertensive status (1694); obese status (n = 1694); sex (n=1694) in the entire cohort is presented.

Supplementary Table 6. The classification accuracy of gut microbiota for estimating metabolic disease indicators using a Random Forest model at each study site

	Diabetes Status	Glucose Status	HTN Status	Sex
Ghana	0.69	0.55	0.63	0.76
South Africa	0.57	0.78	0.54	0.74
Jamaica	0.66	0.58	0.56	0.63
Seychelles	0.54	0.56	0.48	0.75
USA	0.53	0.59	0.47	0.66

Classification accuracy is presented as average area under the curve (AUC) values.
HTN = Hypertension.

Supplementary Table 7. Confusion matrix table showing the average prediction accuracy of gut microbiota for estimating metabolic disease indicators in the Ghanaian cohort using a Random Forest model

	Predicted value		
	Diabetic	Non-Diabetic	Overall accuracy
Diabetic	0	1	
Non-Diabetic	0	1	
Overall accuracy			0.93
Baseline accuracy			0.93
Accuracy ratio			1
	Elevated glucose	Normal glucose	Overall accuracy
Elevated glucose	0	1	
Normal glucose	0	1	
Overall accuracy			0.72
Baseline accuracy			0.72
Accuracy ratio			1
	Hypertensive	Non-Hypertensive	Overall accuracy
Hypertensive	0.24	0.76	
Non-Hypertensive	0.05	0.95	
Overall accuracy			0.75
Baseline accuracy			0.72
Accuracy ratio			1.05
	Obese	Non-Obese	Overall accuracy
Obese	0	1	
Non-Obese	0.02	0.98	
Overall accuracy			0.72
Baseline accuracy			0.73
Accuracy ratio			0.98
	Female	Male	Overall accuracy
Female	0.9	0.1	
Male	0.8	0.2	
Overall accuracy			0.67
Baseline accuracy			0.67
Accuracy ratio			1

Supplementary Table 8. Confusion matrix table showing the average prediction accuracy of gut microbiota for estimating metabolic disease indicators in the South African cohort using a Random Forest model

	Predicted value		
	Diabetic	Non-Diabetic	Overall accuracy
Diabetic	0	1	
Non-Diabetic	0	1	
Overall accuracy			0.96
Baseline accuracy			0.96
Accuracy ratio			1
	Elevated glucose	Normal glucose	Overall accuracy
Elevated glucose	0	1	
Normal glucose	0	1	
Overall accuracy			0.92
Baseline accuracy			0.92
Accuracy ratio			1
	Hypertensive	Non-Hypertensive	Overall accuracy
Hypertensive	0.16	0.84	
Non-Hypertensive	0.25	0.75	
Overall accuracy			0.51
Baseline accuracy			0.59
Accuracy ratio			0.86
	Obese	Non-Obese	Overall accuracy
Obese	0.14	0.86	
Non-Obese	0.15	0.85	
Overall accuracy			0.59
Baseline accuracy			0.63
Accuracy ratio			0.94
	Female	Male	Overall accuracy
Female	0.86	0.14	
Male	0.58	0.42	
Overall accuracy			0.68
Baseline accuracy			0.59
Accuracy ratio			1.16

Supplementary Table 9. Confusion matrix table showing the average prediction accuracy of gut microbiota for estimating metabolic disease indicators in the Jamaican cohort using a Random Forest model

	Predicted label		
	Diabetic	Non-Diabetic	Overall accuracy
Diabetic	0	1	
Non-Diabetic	0	1	
Overall accuracy			0.9
Baseline accuracy			0.9
Accuracy ratio			1
	Elevated glucose	Normal glucose	Overall accuracy
Elevated glucose	0	1	
Normal glucose	0	1	
Overall accuracy			0.8
Baseline accuracy			0.8
Accuracy ratio			1
	Hypertensive	Non-Hypertensive	Overall accuracy
Hypertensive	0.92	0.08	
Non-Hypertensive	0.84	0.16	
Overall accuracy			0.58
Baseline accuracy			0.55
Accuracy ratio			1.05
	Obese	Non-Obese	Overall accuracy
Obese	0.41	0.59	
Non-Obese	0.36	0.64	
Overall accuracy			0.53
Baseline accuracy			0.53
Accuracy ratio			1
	Female	Male	Overall accuracy
Female	0.91	0.01	
Male	0.88	0.12	
Overall accuracy			0.65
Baseline accuracy			0.66
Accuracy ratio			0.98

Supplementary Table 10. Confusion matrix table showing the average prediction accuracy of gut microbiota for estimating metabolic disease indicators in the Seychellois cohort using a Random Forest model

	Predicted label		
	Diabetic	Non-Diabetic	Overall accuracy
Diabetic	0	1	
Non-Diabetic	0	1	
Overall accuracy			0.83
Baseline accuracy			0.83
Accuracy ratio			1
	Elevated glucose	Normal glucose	Overall accuracy
Elevated glucose	0.53	0.47	
Normal glucose	0.53	0.47	
Overall accuracy			0.5
Baseline accuracy			0.52
Accuracy ratio			0.97
	Hypertensive	Non-Hypertensive	Overall accuracy
Hypertensive	0.68	0.32	
Non-Hypertensive	0.82	0.18	
Overall accuracy			0.47
Baseline accuracy			0.58
Accuracy ratio			0.82
	Obese	Non-Obese	Overall accuracy
Obese	0	1	
Non-Obese	0.03	0.97	
Overall accuracy			0.59
Baseline accuracy			0.61
Accuracy ratio			0.98
	Female	Male	Overall accuracy
Female	0.86	0.14	
Male	0.52	0.48	
Overall accuracy			0.7
Baseline accuracy			0.56
Accuracy ratio			1.24

Supplementary Table 11. Confusion matrix table showing the average prediction accuracy of gut microbiota for estimating metabolic disease indicators in the US cohort using a Random Forest model

	Predicted label		
	Diabetic	Non-Diabetic	Overall accuracy
Diabetic	0	1	
Non-Diabetic	0	1	
Overall accuracy			0.8
Baseline accuracy			0.8
Accuracy ratio			1
	Elevated glucose	Normal glucose	Overall accuracy
Elevated glucose	0	1	
Normal glucose	0	1	
Overall accuracy			0.77
Baseline accuracy			0.77
Accuracy ratio			1
	Hypertensive	Non-Hypertensive	Overall accuracy
Hypertensive	1	1	
Non-Hypertensive	0	0	
Overall accuracy			0.69
Baseline accuracy			0.69
Accuracy ratio			1
	Obese	Non-Obese	Overall accuracy
Obese	0.9	0.1	
Non-Obese	0.77	0.23	
Overall accuracy			0.66
Baseline accuracy			0.64
Accuracy ratio			1.03
	Female	Male	Overall accuracy
Female	0.93	0.07	
Male	0.7	0.3	
Overall accuracy			0.72
Baseline accuracy			0.67
Accuracy ratio			1.07

Supplementary Table 12. Weight adjusted fecal SCFA levels by country

WOMEN					
	Ghana<br>n=254	South Africa<br>n=228	Jamaica<br>n=263	Seychelles<br>n=196	US<br>n=213
**PROPIONATE (UG/G)**	30.4 ± 18.0	11.6 ± 6.9	11.5 ± 8.5	14.5 ± 8.5	9.1 ± 6.0
**BUTYRATE (UG/G)**	22.1 ± 11.8	10.6 ± 6.6	8.5 ± 6.8	7.0 ± 5.4	9.0 ± 6.5
**ACETATE (UG/G)**	61.3 ± 22.8	15.6 ± 7.4	24.4 ± 13.3	40.9 ± 25.4	16.8 ± 11.1
**TOTAL SCFA (UG/G)**	115.5 ± 45.0	39.5 ± 19.0	46.0 ± 26.3	63.8 ± 35.1	36.4 ± 21.5
**MEN**					
	Ghana<br>n=117	South Africa<br>n=171	Jamaica<br>n=133	Seychelles<br>n=164	US<br>n=107
**PROPIONATE (UG/G)**	34.3 ± 15.9	18.6 ± 13.1	18.3 ± 13.2	21.0 ± 13.9	13.1 ± 9.9
**BUTYRATE (UG/G)**	23.1 ± 14.2	14.5 ± 9.0	13.2 ± 10.1	8.6 ± 4.7	11.3 ± 6.8
**ACETATE (UG/G)**	68.9 ± 22.5	22.1 ± 9.3	27.0 ± 13.3	42.2 ± 17.2	22.3 ± 13.7
**TOTAL SCFA (UG/G)**	128.0 ± 44.2	57.3 ± 27.9	60.3 ± 30.1	72.8 ± 32.0	48.4 ± 27.3

Data are presented as mean ± standard deviation. Source data are provided as a Source Data file.

Supplementary Table 13. Total fecal SCFA by adiposity status

NON-OBESE					
	Ghana<br>n=254	South Africa<br>n=228	Jamaica<br>n=263	Seychelles<br>n=196	US<br>n=213
**PROPIONATE (UG/G)**	34.1 ± 18.3	16.8 ± 11.8	16.6 ± 13.0	19.2 ± 13.2	12.7 ± 9.6
**BUTYRATE (UG/G)**	23.8 ± 13.2	14.3 ± 8.6	12.2 ± 9.7	8.4 ± 5.9	11.1 ± 7.4
**ACETATE (UG/G)**	69.3 ± 22.4	21.4 ± 9.2	28.3 ± 15.3	45.9 ± 24.1	23.1 ± 14.2
**TOTAL SCFA (UG/G)**	129.1 ± 45.0	54.6 ± 26.3	59.0 ± 32.6	74.8 ± 37.6	48.9 ± 28.4
**OBESE**					
	Ghana<br>n=89	South Africa<br>n=141	Jamaica<br>n=178	Seychelles<br>n=132	US<br>n=201
**PROPIONATE (UG/G)**	24.9 ± 12.4	10.7 ± 6.3	10.3 ± 5.9	14.6 ± 8.1	9.1 ± 6.1
**BUTYRATE (UG/G)**	18.7 ± 10.2	8.7 ± 5.0	7.7 ± 5.6	6.6 ± 3.3	9.0 ± 6.1
**ACETATE (UG/G)**	48.7 ± 16.9	13.2 ± 4.9	21.6 ± 9.5	34.9 ± 16.8	16.0 ± 10.1
**TOTAL SCFA (UG/G)**	93.9 ± 33.9	34.1 ± 14.5	40.9 ± 18.0	57.1 ± 24.0	35.4 ± 19.9

Data are presented as mean ± standard deviation. Source data are provided as a Source Data file.

Supplementary Table 14. Associations between concentrations of the different types of fecal short chain fatty acids (SCFAs) with alpha diversity and BMI within each country

		Ghana		South Africa		Jamaica		Seychelles		USA	
		r	p	r	p	r	p	r	p	r	p
BMI	Total SCFA	0.01	0.82	-0.02	0.73	0.08	0.16	0.02	0.76	0.07	0.20
Shannon	Total SCFA	-0.04	0.44	-0.11	0.05	-0.01	0.03	-0.10	0.06	0.06	0.27
Observed		-0.12	0.03	-0.19	0.00	-0.29	0.00	-0.20	0.00	-0.17	0.00
Shannon		0.05	0.33	-0.03	0.57	-0.21	0.00	-0.11	0.03	0.04	0.51
Observed	Acetate	-0.01	0.84	-0.07	0.17	-0.35	0.00	-0.19	0.00	-0.18	0.00
Shannon		-0.10	0.06	-0.14	0.01	0.02	0.77	-0.05	0.36	0.08	0.19
Observed	Butyrate	-0.20	0.00	-0.25	0.00	-0.14	0.01	-0.10	0.05	-0.10	0.07
Shannon		-0.14	0.01	-0.16	0.00	0.06	0.28	-0.10	0.05	0.03	0.61
Observed	Propionate	-0.18	0.00	-0.26	0.00	-0.13	0.02	-0.23	0.00	-0.21	0.00
Shannon		0.41	0.00	0.14	0.01	0.19	0.00	0.21	0.00	0.12	0.04
Observed	Valerate	0.46	0.00	0.24	0.00	0.32	0.00	0.32	0.00	0.19	0.00

r = Spearman correlation, BMI = body mass index, SCFA = short chain fatty acids

Supplementary Table 15: The effect of short chain fatty acids on obesity through the mediating effects of Shannon diversity in the entire cohort

	Estimate	95% CI lower	95% CI upper	p value
ACME	0	0	0	1
ADE	-0.0003	-0.0004	0	< 2e-16
Total effect	-0.0003	-0.0004	0	< 2e-16
Prop. mediated	0	0	0	1

Boostrap 95% confidence interval with the percentile method (Mediation test, two-sided). Exact p-values are provided. n= 1655. ACME = Average Causal Mediation Effects; ADE = Average Direct Effects; Prop. mediated = Proportion mediated. CI = Confidence Interval

Supplementary Fig 1. Differentially abundant taxa among countries

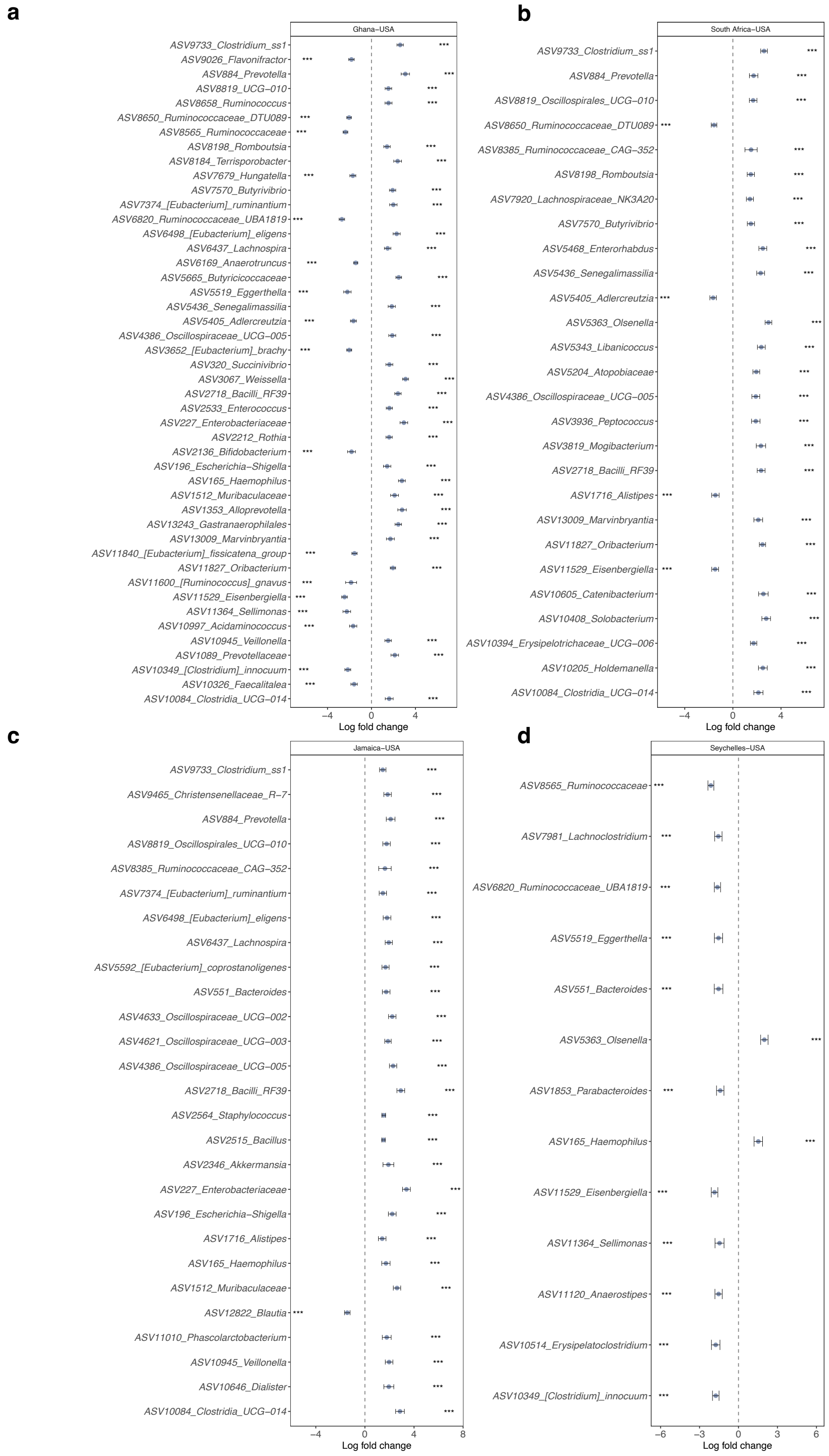

(a-d) Differentially abundant taxa among countries with US as the reference. ANCOM-BC analyses adjusted for BMI, age, and sex. Data are presented by effect size (log fold change) with 95% confidence interval (CI) calculated from the beta coefficient (effect size) and standard errors estimated from the ANCOM-BC log-linear model (two-sided; FDR-adjusted). Colored dot indicates effect size (log fold change) and whiskers indicate 95% CI. ASVs with log fold change >1.4 are shown for country. FDR-adjusted ($q < 0.05$) effect sizes are indicated by *, ** and ***, corresponding to $q < 0.05$, <0.01 and <0.001 respectively. The exact *p*-values are available in the source data file. n = 1694 samples. Source data are provided as a Source Data file. BMI = Body Mass Index. ANCOM-BC = Analysis of Compositions of Microbiomes with Bias Correction. FDR= False Discovery Rate.

Supplementary Fig. 2. Heatmap representations of the 30 most predictive microbial features identified by Random Forest analysis

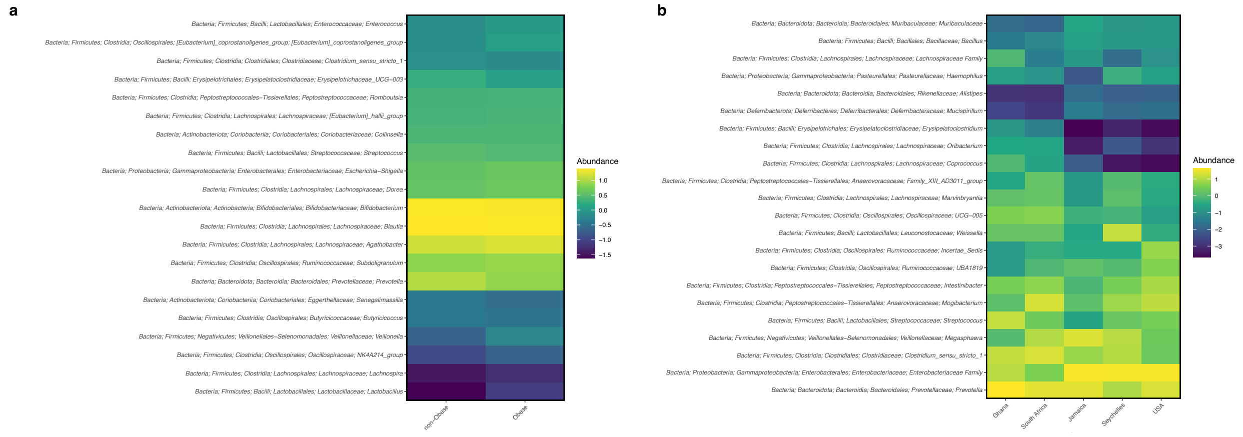

a Heatmap of predictive microbial features classifying countries. **b** Heatmap of predictive microbial features classifying obese and non-obese groups. Microbial features in rows. Source data are provided as a Source Data file. n= 1694 samples

Supplementary Fig. 3. Variation in predicted PICRUSt2 MetaCyc pathways

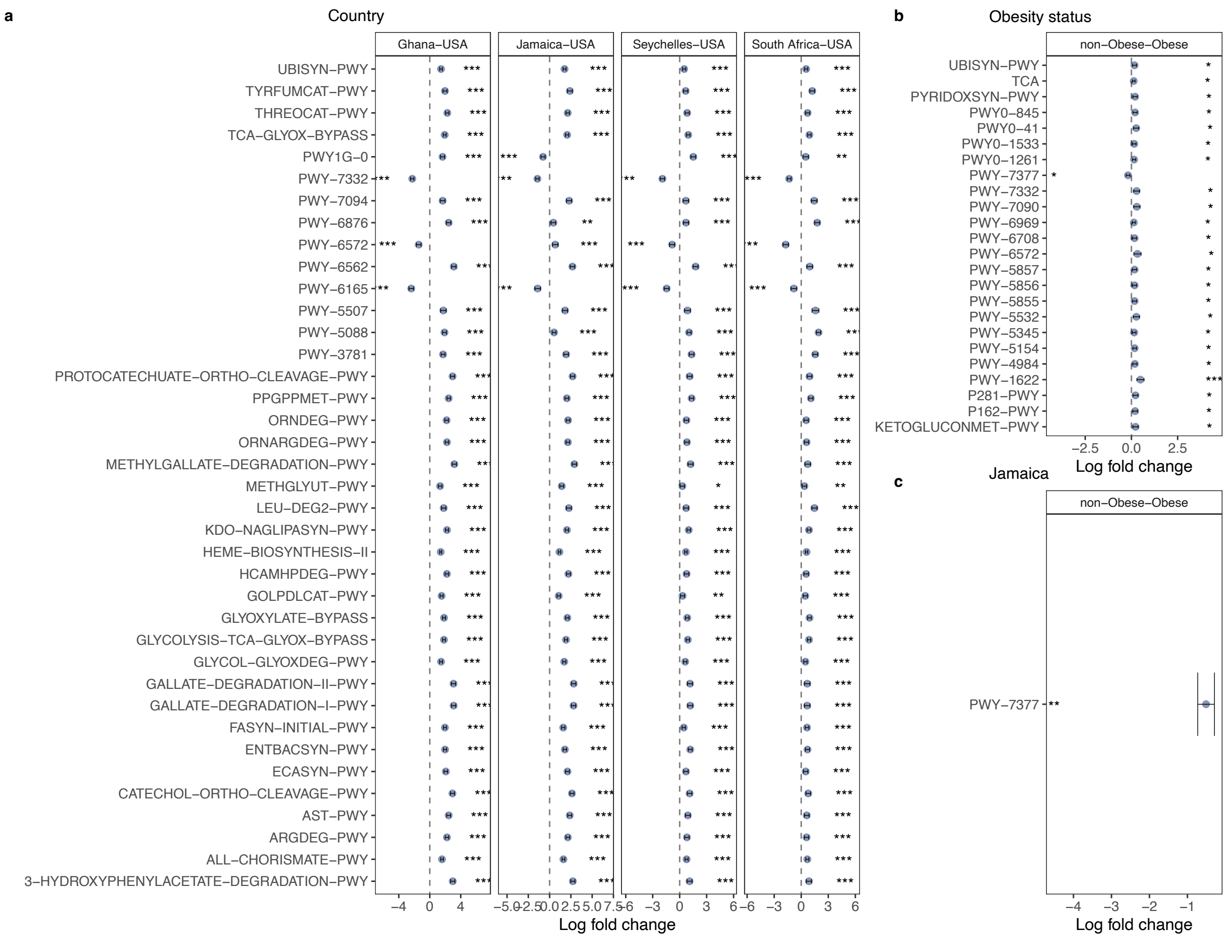

a Differentially abundant predicted MetaCyc pathways among countries with US as the reference (n = 1694). **b** Differentially abundant predicted MetaCyc pathways between obese and non-obese group in entire cohort (n = 1694). **c** Differentially abundant predicted MetaCyc pathways between obese and non-obese group within Jamaica (n = 361). ANCOM-BC analyses adjusted for BMI, age, sex and country. Data are presented by effect size (log fold change) with 95% confidence interval (CI) calculated from the beta coefficient (effect size) and standard errors estimated from the ANCOM-BC log-linear model (two-sided; FDR-adjusted). Colored dot indicates effect size (log fold change) and whiskers indicate 95% CI. Representative predicted pathways with log fold change >1.4 in at least one group are shown for country. FDR-adjusted ($q < 0.05$) effect sizes are indicated by *, ** and ***, corresponding to $q < 0.05$, <0.01 and <0.001 respectively. The exact *p*-values are available in the source data file. Source data are provided as a Source Data file. BMI = Body Mass Index. ANCOM-BC = Analysis of Compositions of Microbiomes with Bias Correction. FDR= False Discovery Rate

Supplementary Fig. 4. Variation in predicted PICRUSt2 KEGG orthology in butanoate metabolism pathway

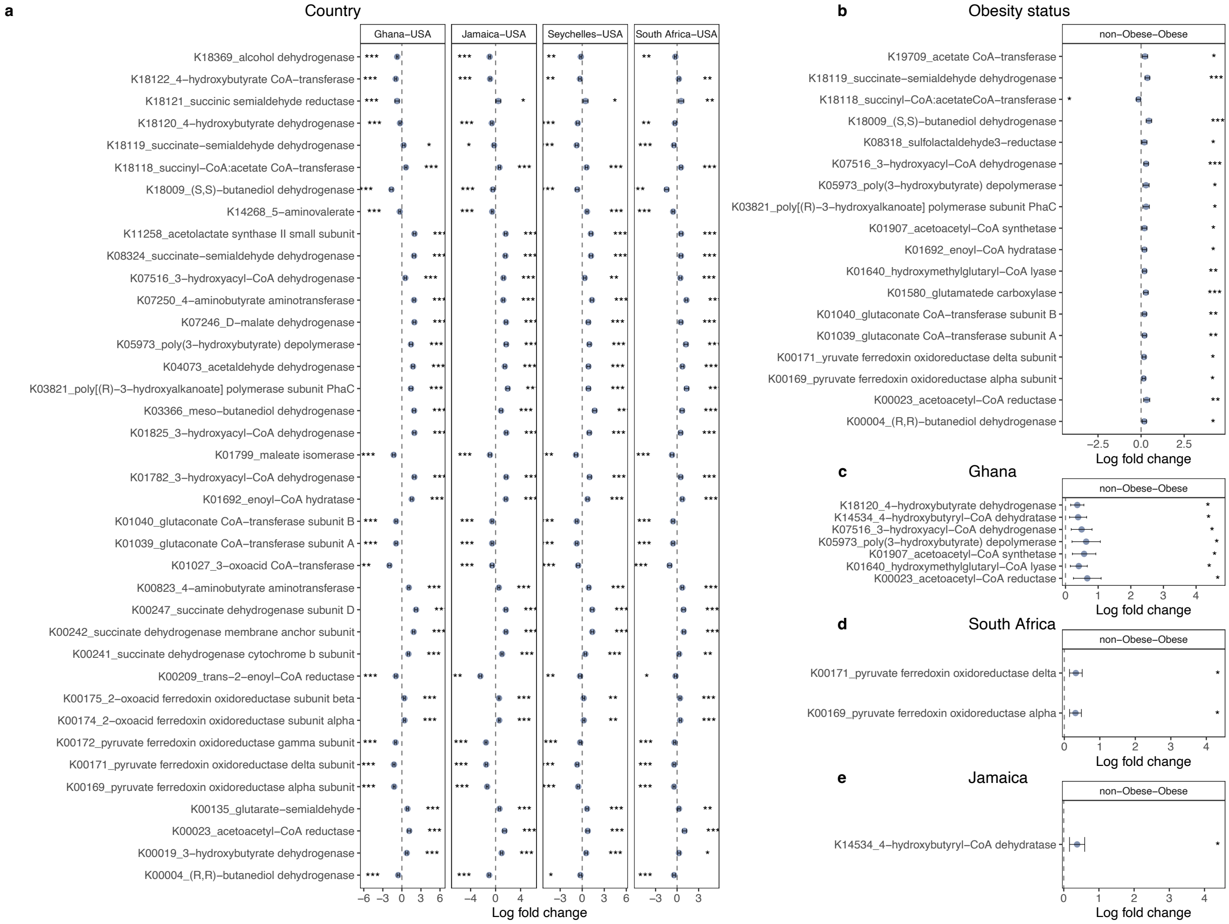

(a) Differentially abundant predicted butanoate KO among countries with US as the reference (n= 1694). (**b**) Differentially abundant butanoate KO between obese and non-obese group in entire cohort. Differentially abundant butanoate KO between obese and non-obese group within (**c**) Ghana (n = 328); (**d**) South Africa (n = 374); (**e**) Jamaica (n = 361). ANCOM-BC analyses adjusted for BMI, age, sex and country using ANCOM-BC. Data are presented by effect size (log fold change) with 95% confidence interval (CI) calculated from the beta coefficient (effect size) and standard errors estimated from the ANCOM-BC log-linear model (two-sided; FDR-adjusted). Colored dot indicates effect size (log fold change) and whiskers indicate 95% CI. Representative predicted pathways with log fold change >1.4 in at least one group are shown for country. FDR-adjusted (q < 0.05) effect sizes are indicated by *, ** and ***, corresponding to *q* < 0.05, <0.01 and <0.001 respectively. The exact *p*-values are available in the source data file. Source data are provided as a Source Data file. KO = KEGG orthology. BMI = Body Mass Index. ANCOM-BC = Analysis of Compositions of Microbiomes with Bias Correction. FDR= False Discovery Rate

Supplementary Fig. 5. Variation in predicted PICRUSt2 KEGG orthology in LPS biosynthesis pathway

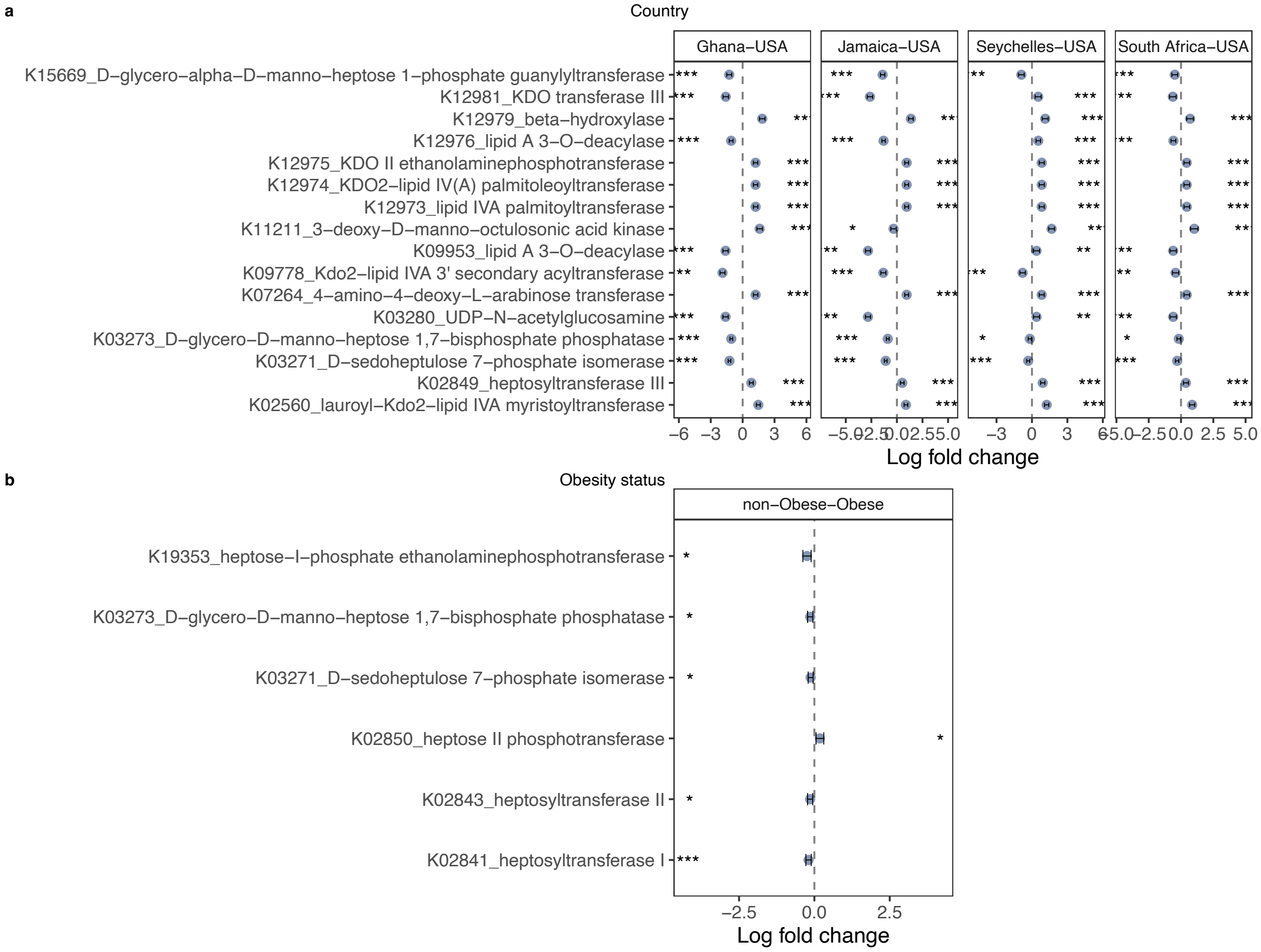

(**a**) Differentially abundant predicted LPS KO among countries with US as the reference. (**b**) Differentially abundant LPS KO between obese and non-obese group in entire cohort. ANCOM-BC analyses adjusted for BMI, age, sex and country. Data are presented by effect size (log fold change) with 95% confidence interval (CI) calculated from the beta coefficient and standard errors estimated from the ANCOM-BC log-linear model (two-sided; FDR-adjusted). Colored dot indicates effect size (log fold change) and whiskers indicate 95% CI. Representative predicted pathways are shown for country. FDR-adjusted ($q < 0.05$) effect sizes are indicated by *, ** and ***, corresponding to $q < 0.05$, <0.01 and <0.001 respectively. The exact *p*-values are available in the source data file. n= 1694 samples. Source data are provided as a Source Data file. BMI = Body Mass Index. KO = KEGG orthology. LPS = Lipopolysaccharides. ANCOM-BC = Analysis of Compositions of Microbiomes with Bias Correction. FDR= False Discovery Rate.

Supplementary Figure 6. The utility of SCFAs to predict obesity using an XGBoost machine learning model.

	Total SCFA	Acetate	Butyrate	Propionate	Valerate
Entire cohort	0.55	0.53	0.52	0.53	0.51
Ghana	0.51	0.53	0.46	0.49	0.60
South Africa	0.51	0.51	0.51	0.55	0.54
Jamaica	0.56	0.56	0.51	0.49	0.55
Seychelles	0.44	0.53	0.51	0.53	0.40
USA	0.48	0.52	0.51	0.51	0.48

The model was tested with 10-fold cross validation in 100 repetitions and the average AUC (area under the receiver operating characteristic curve) for the entire cohort or at each site is presented. Color gradient (red to deep green) represents increasing average AUC values. red: 0.40 – 0.43; light red: 0.44 – 0.46; orange: 0.47 – 0.49; yellow: 0.50 – 0.52; light green: 0.53 – 0.54; green: 0.55 – 0.56; deep green: 0.60. SCFAs = Short chain fatty acids.
