# Supplementary material for: Gut microbiota and fecal short chain fatty acids differ with adiposity and country of origin: the METS-microbiome study
Source: Nat Commun. 2023 Aug 24;14:5160. doi: 10.1038/s41467-023-40874-x (PMC10449869; doi:10.1038/s41467-023-40874-x)
Supplement: Supplementary file 3 — Reporting Summary [file 41467_2023_40874_MOESM3_ESM.pdf]

nature portfolio

Corresponding author(s): Jack Gilbert; Lara Dugas

Last updated by author(s): 8/4/2023

Reporting Summary

Nature Portfolio wishes to improve the reproducibility of the work that we publish. This form provides structure for consistency and transparency in reporting. For further information on Nature Portfolio policies, see our Editorial Policies and the Editorial Policy Checklist.

Statistics

For all statistical analyses, confirm that the following items are present in the figure legend, table legend, main text, or Methods section.

n/a	Confirmed	
☐	☒	The exact sample size (*n*) for each experimental group/condition, given as a discrete number and unit of measurement
☐	☒	A statement on whether measurements were taken from distinct samples or whether the same sample was measured repeatedly
☐	☒	The statistical test(s) used AND whether they are one- or two-sided<br>*Only common tests should be described solely by name; describe more complex techniques in the Methods section.*
☐	☒	A description of all covariates tested
☐	☒	A description of any assumptions or corrections, such as tests of normality and adjustment for multiple comparisons
☐	☒	A full description of the statistical parameters including central tendency (e.g. means) or other basic estimates (e.g. regression coefficient) AND variation (e.g. standard deviation) or associated estimates of uncertainty (e.g. confidence intervals)
☐	☒	For null hypothesis testing, the test statistic (e.g. *F*, *t*, *r*) with confidence intervals, effect sizes, degrees of freedom and *P* value noted<br>*Give P values as exact values whenever suitable.*
☒	☐	For Bayesian analysis, information on the choice of priors and Markov chain Monte Carlo settings
☒	☐	For hierarchical and complex designs, identification of the appropriate level for tests and full reporting of outcomes
☐	☒	Estimates of effect sizes (e.g. Cohen's *d*, Pearson's *r*), indicating how they were calculated

Our web collection on statistics for biologists contains articles on many of the points above.

Software and code

Policy information about availability of computer code

Data collection	No software was used for data collection. All information are provided in the methods section
Data analysis	All data processing and analyses were conducted using publicly available tools. Details are available in the methods section.<br>Raw sequencing reads were processed in Qiita (study ID:13512) and Qiime2 2021.11. Analysis codes are available at https://doi.org/10.6084/m9.figshare.23542395.v1. The phyloseq package 1.38.0 were used to merge the ASV counts, taxonomy assignments, phylogenetic tree, and metadata. Statistical analysis and data visualization were performed in R version 4.1.1 using the following freely available packages: ANCOMBC v1.2.2, ggplot2 v 3.3.6, vegan v 2.6.2, phyloseq v 1.38.0, microbiome v 1.19.1, microbiomeutilities v 1.00.16, gghalves v 0.1.3, qiime2R v0.99.6, tidyverse v1.3.1, reshape v0.8.8, microViz v 0.9.4, cowplot v1.1.1, picante v1.8.2, reshape v 0.8.8, lme4 v1.1-31, xGBoost v1.7.5.1, mediation v4.5.0, RColorBrewer v1.1-3, gtable v 0.3, Biostrings v2.62.0, biomformat v1.22.0, rstatix 0.7.0, patchwork 1.1.1, readr v2.1.2. PICRUSt2 v2.5.1 was installed in python v 3.7.4.<br>Codes for implementation of PICRUSt2 and differential abundance analyses are available at https://github.com/picrust/picrust2 and https://github.com/FrederickHuangLin/ANCOMBC respectively

For manuscripts utilizing custom algorithms or software that are central to the research but not yet described in published literature, software must be made available to editors and reviewers. We strongly encourage code deposition in a community repository (e.g. GitHub). See the Nature Portfolio guidelines for submitting code & software for further information.

Data

Policy information about availability of data

All manuscripts must include a data availability statement. This statement should provide the following information, where applicable:

- Accession codes, unique identifiers, or web links for publicly available datasets
- A description of any restrictions on data availability
- For clinical datasets or third party data, please ensure that the statement adheres to our policy

All 16S rRNA gene sequence data have been deposited at the European Bioinformatics Institute site under the accession code (https://www.ebi.ac.uk/ena/browser/view/PRJEB63378). Additionally, sequencing data and processed tables are available through QIITA under study identifier 13512. The SILVA 16S rRNA database used for alignment is available at https://data.qiime2.org/2022.2/common/silva-138-99-515-806-nb-classifier.qza. Taxonomy was assigned to ASVs using the SILVA ribosomal RNA gene database (version 138). ASVs were inserted into the Greengenes 13.8 99% identity tree with SATé-enabled phylogenetic placement (SEPP) to generate a phylogenetic tree. The KEGG and MetaCyc Databases are available at https://www.genome.jp/kegg/ and https://metacyc.org/ respectively. The data and analyses generated in this study are available within the paper, Supplementary Information and Source data files provided with this paper.

Research involving human participants, their data, or biological material

Policy information about studies with human participants or human data. See also policy information about sex, gender (identity/presentation), and sexual orientation and race, ethnicity and racism.

Reporting on sex and gender	We collected information on self-reported sex and have reported it in the participants characteristics table. Some analysis were separately done for sex while in other analyses sex was included as a covariate.
Reporting on race, ethnicity, or other socially relevant groupings	The METS Microbiome participants are populations of African origin in 5 different countries and this information was by self-report. Country was included into the analyses as a covariate
Population characteristics	In 2018-2019, METS-Microbiome recruited participants 2,085 participants (~60% women) ages 35-55 years old from Ghana, South Africa, Jamaica, Seychelles, and US, representing the spectrum of the 'epidemiologic transition' with Ghana and the USA representing the two extremes. Populations sampled represent a range of social and economic development as defined by the United Nations Human Development Index http://hdr.undp.org/en/content/human-development-index-hdi. Participants were excluded if they self-reported an infectious disease, including HIV-positivity, pregnancy, antibiotic usage within 3 months, breast-feeding or any condition which prevented the individual from participating in normal physical activities. A description of the METS protocol has been published (Luke A, et al. 2011. BMC Public Health 11:927; Dugas L, et al. 2018. BMC Public Health 18 (1): 978). Several clinical data including age, sex, BMI, blood pressure measurements and fasted blood glucose were collected. Detailed population characteristics can be found in the manuscript.
Recruitment	From 2018-2019, the METS-Microbiome study recruited 2,085 participants (~60% women) ages 35-55 years old from five different sites (Ghana, South Africa, Jamaica, Seychelles, and US). Of these participants, 1,249 have been followed on a yearly basis since 2010 under the parent METS study. Recruitment for this study has been described in Dugas et al 2018 BMC Public Health 18 (1): 978. A detailed description about study population and covariates included in analyses are provided in the methods section and Table 1 of the manuscript.
Ethics oversight	METS-Microbiome was approved by the Institutional Review Board of Loyola University Chicago, IL, US; the Committee on Human Research Publication and Ethics of Kwame Nkrumah University of Science and Technology, Kumasi, Ghana; the Research Ethics Committee of the University of Cape Town, South Africa; the Board for Ethics and Clinical Research of the University of Lausanne, Switzerland; and the Ethics Committee of the University of the West Indies, Kingston, Jamaica.

Note that full information on the approval of the study protocol must also be provided in the manuscript.

Field-specific reporting

Please select the one below that is the best fit for your research. If you are not sure, read the appropriate sections before making your selection.

☒ Life sciences ☐ Behavioural & social sciences ☐ Ecological, evolutionary & environmental sciences

For a reference copy of the document with all sections, see nature.com/documents/nr-reporting-summary-flat.pdf

Life sciences study design

All studies must disclose on these points even when the disclosure is negative.

Sample size	A full description of the METS cohort and sampling strategy is provided in the manuscript and has also been published (Luke A, et al. 2011. BMC Public Health 11:927; Dugas et al 2018 BMC Public Health 18 (1): 978). Sample size was calculated based on the association between SCFAs and body fat (BF). Because BF varies by site, we used separate models across sites. To detect a R^2 of 0.06 with 80% power we needed 199 participants/site.
Data exclusions	Exclusion and inclusion criteria are described in the methods. Participants were excluded from participating in the original METS study if they

	self-reported being persons with an infectious disease including HIV, being pregnant, breast-feeding, using antibiotics within 3 months or having any condition which prevented the individual from participating in normal physical activities. Data points that were not answered or were unclear were excluded while retaining the participant and the reliable data and sample. 16S rRNA amplicon sequencing depth of below 6000 reads and ASVs with less than ten reads in the entire dataset were excluded to provide robust diversity analysis. For differential abundance, random forest, correlation and PICRUSt2 analyses, samples with less than 10,000 reads and ASVs with fewer than 50 reads in total across all samples and/or were present in less than 2% of samples were excluded.
Replication	Our current study is observational, and it is possible that our findings are restricted to our population cohort. Nevertheless, we recruited participants from 5 geographically diverse countries. Other measures taken for reproducibility included participants enrollment by the study coordinators who were not involved in either data processing or analyses, double entering of metadata by two independent individuals, errors identified were corrected. Similarly, bioinformatics and statistical analyses were conducted independently by two different individuals.
Randomization	This is a population based study. There are no conditions to randomize study participants.
Blinding	There was no control or intervention arm so blinding was not applicable. However, Data collection and analysis were done by different researchers. DNA extraction and sequencing was conducted at a microbiome and sequencing core that were blind to status of the samples. SCFA measurements were also conducted in a mass spectrometry core that were blind to the status of the samples.

Reporting for specific materials, systems and methods

We require information from authors about some types of materials, experimental systems and methods used in many studies. Here, indicate whether each material, system or method listed is relevant to your study. If you are not sure if a list item applies to your research, read the appropriate section before selecting a response.

Materials & experimental systems

n/a	Involved in the study	
☒	☐	Antibodies
☒	☐	Eukaryotic cell lines
☒	☐	Palaeontology and archaeology
☒	☐	Animals and other organisms
☐	☒	Clinical data
☒	☐	Dual use research of concern
☒	☐	Plants

Methods

n/a	Involved in the study	
☒	☐	ChIP-seq
☒	☐	Flow cytometry
☒	☐	MRI-based neuroimaging

Clinical data

Policy information about clinical studies

All manuscripts should comply with the ICMJEguidelines for publication of clinical research and a completedCONSORT checklist must be included with all submissions.

Clinical trial registration	The study has been registered under clinicaltrials.gov. https://clinicaltrials.gov/ct2/show/NCT03378765 NCT03378765
Study protocol	Dugas, R et al. Gut microbiota, short chain fatty acids, and obesity across the epidemiologic transition: the METS-Microbiome study protocol. BMC Public Health. 2018 Aug 6;18(1):978. doi: 10.1186/s12889-018-5879-6.
Data collection	From 2018-2019, the METS-Microbiome study recruited 2,085 participants (~60% women) ages 35-55 years old from five different sites (Ghana, South Africa, Jamaica, Seychelles, and US). Of these participants. Participants were excluded if they self-reported an infectious disease, including HIV-positivity, pregnancy, antibiotic usage within 3 months, breast-feeding or any condition which prevented the individual from participating in normal physical activities.
Outcomes	Primary outcome is changes in microbiome composition and levels of short chain fatty acids (SCFAs) between countries of African origin and obesity status. To measure this, stools samples were sequenced; alpha and beta diversity and differentially abundant taxa were determined between the different cohorts and between obese and non-obese in the entire cohort and separately for each country. Targeted stool metabolomics were performed and the concentrations of each of the 4 SCFAs among the various countries and also between obese and non-obese in the entire cohort were determined. We tested the hypothesis that microbiota diversity will be associated with the urbanization spectrum, SCFA levels and obesity status. Obesity was defined as ≥ 30 kg/m2.<br><br>Secondary outcomes tested how alterations in microbiome composition are associated with other metabolic disease risks. Notably, hypertension was defined as mean systolic/diastolic blood pressure ≥ 130/80 mm Hg or on current treatment. Diabetes was defined as >125mg/dL or current treatment for all sites, except for Ghana as not all participants were fasted overnight. For the Ghanaian site, diabetes was defined as ≥140mg/dL or current treatment according to American Diabetes Association guidelines for random glucose testing.
